# Formation of Ciprofloxacin–Isonicotinic Acid Cocrystal Using Mechanochemical Synthesis Routes—An Investigation into Critical Process Parameters

**DOI:** 10.3390/pharmaceutics14030634

**Published:** 2022-03-13

**Authors:** Maryam Karimi-Jafari, Ahmad Ziaee, Emmet O’Reilly, Denise Croker, Gavin Walker

**Affiliations:** Synthesis & Solid State Pharmaceutical Centre (SSPC), Department of Chemical Sciences, Bernal Institute, University of Limerick, V94 T9PX Limerick, Ireland; maryam.karimijafari@ul.ie (M.K.-J.); emmet.oreilly@ul.ie (E.O.); denisecroker09@gmail.com (D.C.); gavin.walker@ul.ie (G.W.)

**Keywords:** cocrystal, mechanochemical synthesis, hot-melt extrusion, ball milling, grinding, solid state chemistry, continuous manufacturing, design of experiment, green chemistry

## Abstract

The mechanochemical synthesis of cocrystals has been introduced as a promising approach of formulating poorly water-soluble active pharmaceutical ingredients (APIs). In this study, hot-melt extrusion (HME) as a continuous process and grinding and ball milling as batch processes were employed to explore the feasibility of cocrystallization. Ciprofloxacin (CIP) and isonicotinic acid (INCA) were selected as the model API and coformer. CIP–INCA cocrystal was produced in all techniques. It was revealed that higher cocrystal content could be achieved at longer durations of grinding and ball milling. However, milling for more than 10 min led to increased co-amorphous content instead of cocrystal. A design of experiment (DoE) approach was used for deciphering the complex correlation of screw configuration, screw speed, and temperature as HME process parameters and their respective effect on final relative cocrystal yield. Statistical analysis showed that screw configuration, temperature, and their interaction were the most critical factors affecting cocrystallization. Interestingly, screw speed had minimal impact on the relative cocrystallization yield. Cocrystallization led to increased dissolution rate of CIP in phosphate buffer up to 2.5-fold. Overall, this study shed a light on the potential of mechanochemical synthesis techniques with special focus on HME as a continuous process for producing cocrystals.

## 1. Introduction

About 40% of newly marketed drugs have low solubility and 70–90% of drugs in the development pipeline are categorized in the low solubility classification [1]. A study on 812 drug candidates from Pfizer, AstraZeneca, Eli Lilly, and GlaxoSmithKline depicted that poor bioavailability and pharmacokinetic are the third main failure cause of the molecules in phase 1 clinical trials [2]. Traditionally, different solid form modifications of a drug such as metastable polymorph(s), amorphous form, solvate/hydrates, and salt formation have been used for altering the physicochemical properties of APIs. Each of these strategies has its own benefits and challenges.

Metastable polymorphs usually only offer a 1- to 2-fold increase in solubility, while maintaining their processability and shelf-life has been extremely challenging due to their poor physical and chemical stability [3]. Moreover, solvates/hydrates are not always the most desirable solid forms for manipulating physicochemical properties of APIs due to their low thermal stability and minimal impact on solubility [4,5,6]. Additionally, transformation of crystalline APIs to amorphous form potentially leads to improved solubility of poorly water-soluble APIs. However, high recrystallization tendency of amorphous APIs due to their higher free energy and entropy is a significant challenge for their further development [7,8]. Salt formation is a well-established approach for modifying the physicochemical properties of a drug substance. It is only applicable to APIs with ionizable moieties [9]. Cocrystal formation has been introduced as a promising alternative to commonly used solid form control strategies [10,11,12]. It can afford a broad platform for optimizing API properties based on the pharmaceutical development requirements. For instance, cocrystallization of itraconazole as an antifungal drug with very low aqueous solubility by adding succinic, L-malic, and L-tartaric acid as coformer increased its dissolution rate compared with its pure crystalline form [13]. Furthermore, Hickey et al. showed that cocrystallization of carbamazepine and saccharin led to its improved stability [14]. The recent advances in crystal engineering field, regulatory acceptance, and ease of access to a wide range of coformers have paved the way for adaptation of cocrystallization within the pharmaceutical industry [15].

The pharmaceutical industry is facing myriad challenges to overcoming economic limitations, operational complications, and flexible market demands [16,17,18]. Moreover, the patents lifetime of newly developed drug products has been significantly reduced by market globalization. This has caused the need for minimising the development time for new products on one hand and maximising their production throughput on the other hand. Continuous manufacturing (CM) is the answer to the need of maximizing the throughput while maintaining drugs quality. CM has been widely used in industries such as paper, food, plastics, and ceramics, and is gradually being adopted by the pharmaceutical industry as well. Manufacturing cocrystals using techniques such as hot-melt extrusion (HME) has been part of the efforts towards adaptation of CM principles in the pharmaceutical industry [19,20,21].

Ciprofloxacin (CIP), or 1-cyclopropyl-6-fluoro-4-oxo-7-(piperazin-1-yl)-1,4-dihydroquinoline-3-carboxylic acid, is one of the most prescribed APIs globally that was introduced to market for the first time in 1986. It is a broad-spectrum antibiotic of the fluoroquinolone class applied against Gram-positive and Gram-negative bacteria, as well as other microorganisms [22,23]. It has a wide range of applications in bacterial infection treatment, especially respiratory infections, such as sinusitis, pharyngitis, pneumonia, tuberculosis, and bronchitis. Moreover, it is commonly used for treating tissue, bone, and skin infections [24,25].

The dependence of aqueous solubility of CIP on pH has led to its limited application [26]. The ‘U-shape’ pH-solubility profile of CIP shows that its solubility increases at pH values higher than 8.7 (basic pKa) and lower than 6.1 (acidic pKa) [27], with the lowest solubility at neutral pH values. Therefore, CIP has been classified as an intermediate compound with BCS Class II/III classification [28]. In some cases, CIP has been classified as BCS class IV due it low solubility and low permeability, which leads to limited bioavailability [29]. Moreover, ciprofloxaine hydrochloride has been classified as BCS class IV [30]. Several attempts for tackling CIP’s low aqueous solubility issue include salt formation [26,29,31,32,33,34,35,36] and amorphization [23] with less frequent cases of cocrystallization [36,37,38,39,40,41]. As an example, Martinez-Alejo et al. studied the cocrystal of ciprofloxacin and moxifloxacin hydrochloride salts with 4-hydroxybenzoic acid as coformer. Different approaches such as slurry conversion, solvent drop grinding, solvent evaporation, and reaction crystallization have been applied to form the CiHCl-4HBA and MoHCl-4HBA cocrystalline solids. As a result, the aqueous solubility and dissolution rate of the CiHCl-4HBA cocrystal was lower than the starting salt form, but the aqueous solubility of MoHCl-4HBA was improved and higher than its starting material [38].

In addition, it was depicted that cocrystallization of CIP and resorcinol can enhance the dissolution rate of pure CIP at pH 4.0, 5.0, and 5.5. F. J. Caires and co-workers studied the cocrystal formation of CIP with different coformers. They could successfully form the novel cocrystals of CIP with pyrazinoic acid (PZCA) and p-aminobenzoic acid (PABA) in 1:1 stoichiometric ratio. Neat grinding (NG) and liquid-assisted grinding (LAG/ethanol) were used as mechanochemical approaches for cocrystallization. They showed that both NG and LAG methodologies were applicable when forming the cocrystal of CIP–PZCA, but for CIP–PABA, only the LAG approach led to the formation of cocrystal. Moreover, they demonstrated the feasibility of formation of CIP cocrystal with picolinic acid (PCA) in 1:1 molar ratio via LAG, with an increase of 27% in solubility of CIP compared with the pure form of the drug [41]. In another study, nicotinic (NCA) and isonicotinic acid (INCA) were selected as coformer with CIP as API to study cocrystal formation. Mechanochemical methods such as NG and LAG led to the successful cocrystal formation at q 1:1 molar ratio of CIP and NCA and INCA. Two different frequencies (15 Hz and 30 Hz) were used for grinding via ball milling, and based on the results, 30 Hz of grinding frequency contributed more to cocrystallization due to the higher mechanical forces for sample synthesis. Moreover, the solubility of CIP–NCA and CIP–INCA cocrystals was increased up to 20-fold in pure water, while the solubility of CIP–NCA in phosphate buffer pH = 6.8 was improved by about 1.5-fold and for CIP–INCA by 2.5-fold [42]. Traditional batch processes such as grinding and ball milling have been predominantly used for cocrystallization of CIP, despite the strong need for moving towards continuous manufacturing processes. Therefore, hot-melt extrusion was employed to cover the gap in the literature on the application of continuous manufacturing techniques in cocrystallization of the CIP–INCA system.

The aim of this study was to compare three mechanochemical synthesis approaches for cocrystallization of ciprofloxacin, to increase its dissolution rate, and to optimize the processes. Moreover, a design of experiment approach was used to develop continuous manufacturing process and define the most significant critical process parameter for achieving the highest relative cocrystal yield.

## 2. Materials and Methods

### 2.1. Material

Isonicotinic acid (INCA) (CAS no. 55-22-1) with purity ≥99.0% was purchased from Sigma-Aldrich (Dublin, Ireland). Ciprofloxacin (CIP) (CAS no. 85721-33-1) with purity >98% was purchased from Kemprotec (Carnforth, UK). All chemicals were used as received.

### 2.2. Materials Selection Rationale

**Figure 1:**
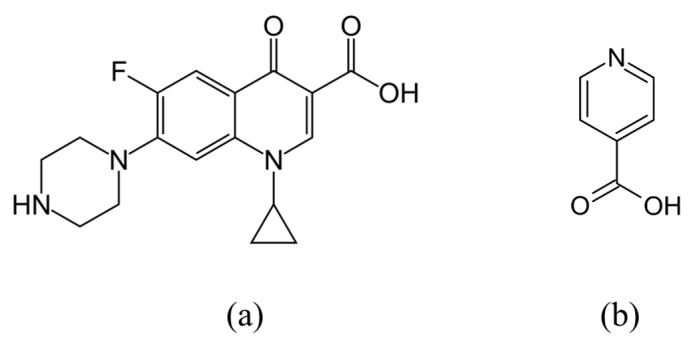
Molecular structure of (**a**) CIP, (**b**) INCA.

The presence of a carboxylic acid group in the CIP structure makes it a proper hydrogen bond donor which facilitates its intermolecular interaction with the coformer. INCA was selected as coformer, which has been reportedly used in combination with other APIs as a hydrogen bond donor and acceptor [43]. Figure 1 shows the molecular structure of CIP and INCA.

### 2.3. Cocrystal Preparation Methods

#### 2.3.1. Neat Grinding

CIP cocrystal with INCA as a coformer was synthesized by a range of mechanochemical techniques including neat grinding of 1:1 stoichiometric ratio of the physical mixture of starting materials (API and coformer) in mortar and pestle. Total amount of 0.5 g of the physical mixture was ground for different time durations (5, 10, 15, 20, and 30 min).

#### 2.3.2. Ball Milling

Milling process was carried out to investigate the cocrystal formation using a ball milling apparatus (MM 400, Retsch, Düsseldorf, Germany) equipped with 10 mL milling jars and one stainless steel grinding ball with diameter of 8.74 mm and weight of 4.06 g. Half a gram of equimolar mixture of CIP–INCA was placed in 10 mL grinding jar. The mixture was milled at 30 Hz for various periods of time (5, 10, 15, 20, and 30 min). The obtained samples were then collected and tested for formation of cocrystals using powder X-ray diffraction (PXRD) and differential scanning calorimetry (DSC).

#### 2.3.3. Hot-Melt Extrusion (HME)

**Figure 2:**
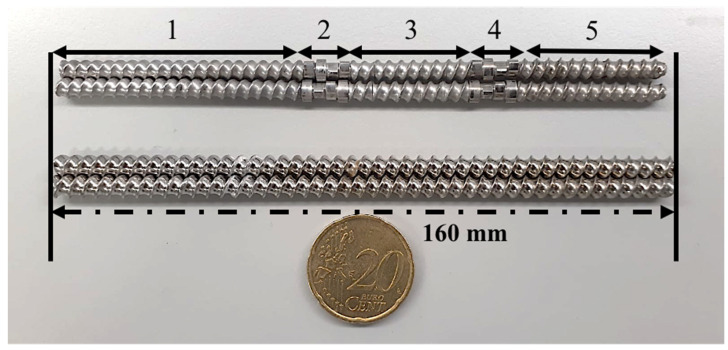
Screw configuration of (**top**) integrated conveying and kneading elements: (**1**) 1st conveying zone, (**2**) 1st kneading zone, (**3**) 2nd conveying zone, (**4**) 2nd kneading zone, (**5**) 3rd conveying zone, (**bottom**) screw with only conveying elements.

A 1:1 molar ratio of CIP–INCA physical mixture was prepared for cocrystallization via hot-melt extrusion. A co-rotating twin screw extruder (Hybrid extruder ZE 5/12, Three-Tec GmbH, Seon, Switzerland) with screw diameter of 5 mm and length-to-diameter ratio of 32:1 (Figure 2) was used. The samples were extruded with two different screw configurations of only conveying and conveying with areas of integrated kneading elements. A 3-zoned barrel with three heating elements was employed, where all the zones were kept at the same temperature. CIP and INCA were mixed (in 0.05 kg batches) in a tubular mixer for 10 min prior to the feeding at a rate of 0.025 kg/h through a gravimetric double screw feeder (ZD 5 FB-C-1M-50 i6000, Three-Tec GmbH, Seon, Switzerland) to the extruder.

Considering the multivariate nature of the HME process, a design of experiment (DoE) was used to evaluate the effect of each of the process factors on the cocrystallization relative yield. Therefore, a three-factor full factorial DoE was used to study the effect of temperature, screw speed, and screw configuration on the relative cocrystallization yield. The DoE identifies the effect of individual factors and their interactions on the studied response. A full factorial DoE is an appropriate choice when fewer than five factors are being investigated, as was the case of this study. Two continuous factors of screw speed and temperature and a categorial factor of screw configuration were considered as effective factors for studying their effects on final relative cocrystal yield. Each continuous factor was studied at three levels, and screw configuration was studied at two levels of conveying and kneading. Statistical analysis of variance (ANOVA) test was performed to determine the significance (*p*-value) and impact (*F*-value) of each main factor as well as their interactions. *F*-value is the indication of statistical significance of the test. The *p*-value for each term tests the null hypothesis that the coefficient associated with a particular term is zero. A small *p*-value (significance level *p*-value < 0.05 is used here) shows that the null hypothesis can be rejected and that the term is significant in adding information to the model. Conversely, a *p*-value > 0.05 indicates that the predictor in question cannot be used to predict changes in the response.

The fitted model was suitable for identifying the most significant factors that affect the final cocrystal yield. Three samples 4, 5, and 14 that were produced at 200 °C and kneading screw configuration were excluded from the analysis due to the extremely low physical yield of the process. The calculated area under the peak of 2θ = 5.37° as the characteristic peak of CIP–INCA cocrystal was correlated with the corresponding relative cocrystal yields. Table 1 shows the studied factors and the corresponding values used for each sample based on the DoE and the calculated relative cocrystal yield.

In order to design the DoE, a set of initial experiments was performed to define the lowest and highest working conditions. Thereby, 100 °C was selected as the minimum temperature because it was the lowest temperature that allowed the transition of powder from the point of entry to the end of the barrel. Moreover, 200 °C was selected as the maximum temperature because at higher temperatures the mixture of the API and coformer transformed to a dark and sticky paste that could not pass through the barrel, and the physical yield was really low. Several considerations were also taken for selecting the range of screw speeds. The lowest range of screw speed was set to 10 rpm, which provided enough residence time for the samples within the barrel. However, in order to determine the effect of screw speed on the cocrystal formation, 100 rpm was selected as the maximum screw speed considering its practicality in industrial environment.

**Figure 3:**
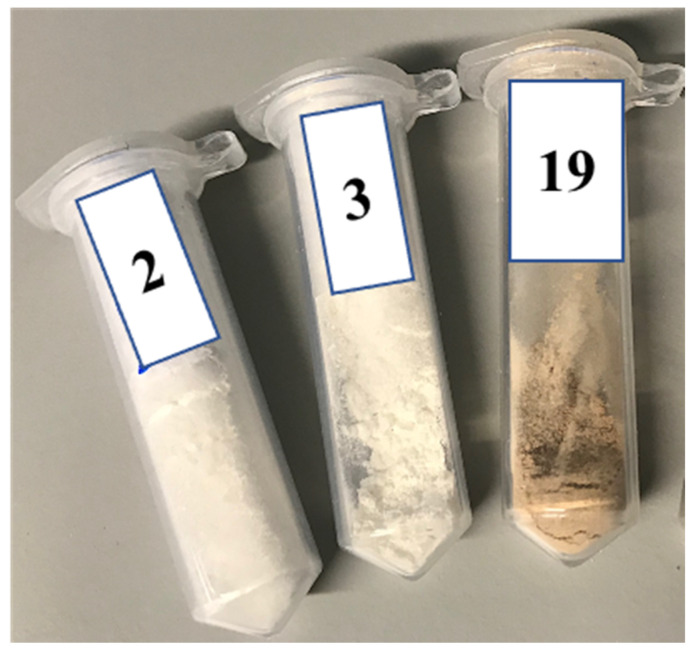
Physical appearance of samples extruded at 100 °C (sample 2), 150 °C (samples 3), and 200 °C (sample 19).

The extruder was operated with a die with a diameter of 1.75 mm in order to collect the final product. The physical appearance of some of the samples at various temperatures is shown in Figure 3. The transition of the colour of powders from white to light brown by increasing the temperature from 100 °C to 200 °C is clearly visible. This signifies the possible transformation of the powder from initial materials to a new compound at higher temperatures.

### 2.4. Powder X-ray Diffraction (PXRD)

The powders were analysed by PXRD to define their crystallinity using a PANalytical X’Pert PRO MRD (PANalytical, Almelo, The Netherlands) with monochromatized Cu Kα radiation (λ = 0.15405 nm). The High Score Plus software was used for running the instrument. The X-ray generator settings were set at 40 kV and 40 mA. The scans were performed over 2θ range of 2.5–40°, with step size of 0.02°/step and step time of 40 s/step.

### 2.5. Differential Scanning Calorimetry (DSC)

The formation of cocrystal compound after HME process was tested via a 214 Polyma DSC (NETZSCH Group, Selb, Germany). A 30 mL/min flow of nitrogen was used as purge gas. Samples of approximately 5.0 mg were weighted into aluminium pans and crimped. Heating rate of 10 °C/min was used to ramp up the temperature in the range of 20–350 °C.

### 2.6. Scanning Electron Microscopy (SEM)

The morphology of the granulated samples produced via HME was investigated by high resolution field emission electron microscopy (SEM, Hitachi SU-70, Tokyo, Japan) operating at 5 kV with 15 mm working distance. To avoid overcharging, the powders were coated with gold–palladium for 2 min with 20 mA current.

### 2.7. Fourier Transform Infrared Spectroscopy (FTIR)

The FTIR spectrum of the granulated samples produced via HME was measured at ambient temperature using a Perkin-Elmer Spectrum 100 FTIR spectrometer (Perkin-Elmer Company Waltham, MA, USA). The spectrum was collected at wavelengths of 4000–450 cm^−1^ using an attenuated total reflection (ATR) accessory with a ZnSe crystal. Samples were placed on the crystal with a pushing arm, and 64 scans were collected for each sample at a resolution of 4.00 cm^−1^.

### 2.8. Dissolution

The dissolution study was performed for sample 10 (highest relative cocrystal yield) following the USP29 procedure for CIP solubility study. A USP II apparatus was employed with paddle rotation speed at 50 rpm for 120 min. The dissolution flasks were filled with 900 mL of prepared 0.1 M phosphate buffer with pH 6.8. Flasks were immersed in water bath to provide fixed and approved temperature at 37 ± 0.5 °C before starting the experiment. The paddles were then assembled and rotated with 50 rpm for 15 min to equilibrate the dissolution media.

Ciprofloxacin shows a U-shape dissolution behaviour as a function of pH with the lowest dissolution rate at pH 6.8, the intestinal fluid pH value. Therefore, this pH was selected in order to be able to observe the potential of cocrystallization approach in addressing the low dissolution rate of CIP. Moreover, CIP shows high dissolution rate at acidic pH values that are not suitable for showing a meaningful difference between CIP and its cocrystal.

After the temperature was equilibrated, the paddles were stopped and a fixed amount of each formulation containing 50 mg equivalent of CIP was added to each dissolution media. The paddles were then immediately rotated at 50 rpm. Samples of 5 mL were collected from the media and filtered using Nylon 0.45 μm filters at specified intervals of 5, 10, 15, 20, 30, 45, 60, 90, and 120 min. The samples were then analysed using a UV-vis spectroscopy setup. For this purpose, the collected aliquots were scanned at 275 nm as λ_max_ for ciprofloxacin to measure the concentration of it within the dissolution media [44].

## 3. Results and Discussion

### 3.1. Grinding

#### 3.1.1. Powder X-ray Diffraction (PXRD)

**Figure 4:**
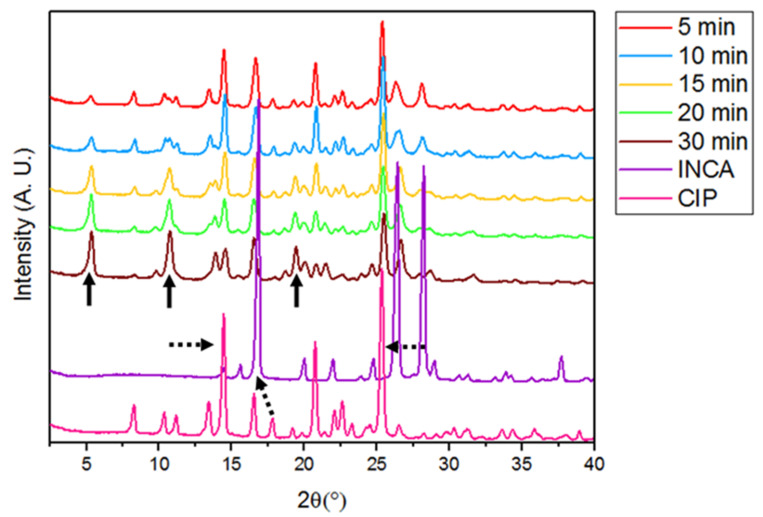
PXRD spectra of ground samples ball milled at 30 Hz for various duration of times.

The PXRD graph of ground samples for different time durations is depicted in Figure 4. Appearance of new peaks was observed even in sample that was ground only for 5 min. Characteristic peaks of cocrystal at 5.4°, 10.6°, and 19.2° could be detected in all of the ground samples regardless of the grinding time. At the same time, the gradual disappearance or decrease in the intensity of main characteristic peaks of initial materials confirmed the transformation of the starting materials to a new cocrystal form. For instance, the peak at 16.9°, which is the peak with highest intensity of INCA, completely disappeared from the spectra of all ground samples. Moreover, the intensity of peaks at 14.5° and 25.3° as the characteristic peaks of CIP gradually decreased over longer grinding durations. Overall, the PXRD spectra confirmed the formation of a new cocrystal. Moreover, the gradual increase in intensity of characteristic peaks of cocrystal at longer grinding times showed the positive correlation between grinding time and cocrystal formation. The longer grinding time led to the formation of new surfaces which increased the chance of formation of new bonds between API and coformer. Cocrystallization mechanisms during neat grinding have been well explained by Friscic and Jones as molecular diffusion, eutectic formation, and cocrystallization mediated by amorphous phase [45]. Therefore, approximately the mechanism of formation of the CIP–INCA cocrystal via neat grinding could involve one, two, or all of the mechanisms, simultaneously.

#### 3.1.2. Differential Scanning Calorimetry (DSC)

**Figure 5:**
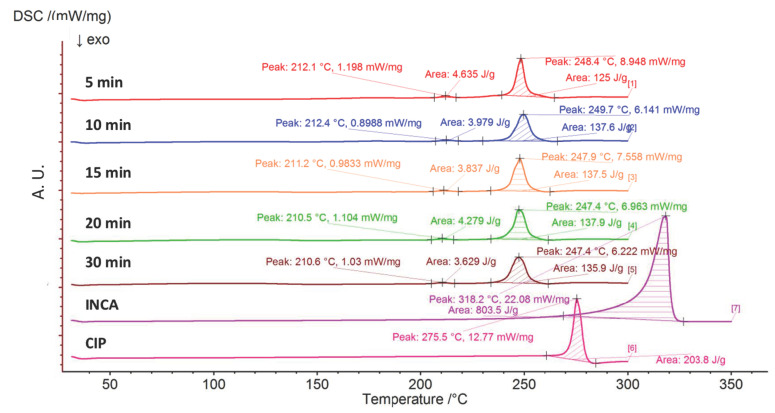
DSC thermogram of ground samples for various duration of times.

To confirm the conclusions derived based on PXRD analysis, samples were analysed using DSC (Figure 5). The melting peak of CIP and INCA as precursors are clearly shown at 275.5 °C and 318.2 °C, respectively. Two new endothermic peaks are visible in all thermograms after grinding, with temperatures lower than melting points of initial materials. One minor peak at approximately 210–212 °C and a major peak at approximately 247–249 °C. The first peak is associated with the crystalline phase transition, while the second endotherm confirms the presence of a major portion of cocrystal. This is in agreement with the binary phase diagram of CIP–INCA cocrystal system that shows the solid to liquid phase transformation at approximately 220 °C, followed by complete melting at temperatures above 240 °C [40].

### 3.2. Ball Milling

#### 3.2.1. Powder X-ray Diffraction (PXRD)

**Figure 6:**
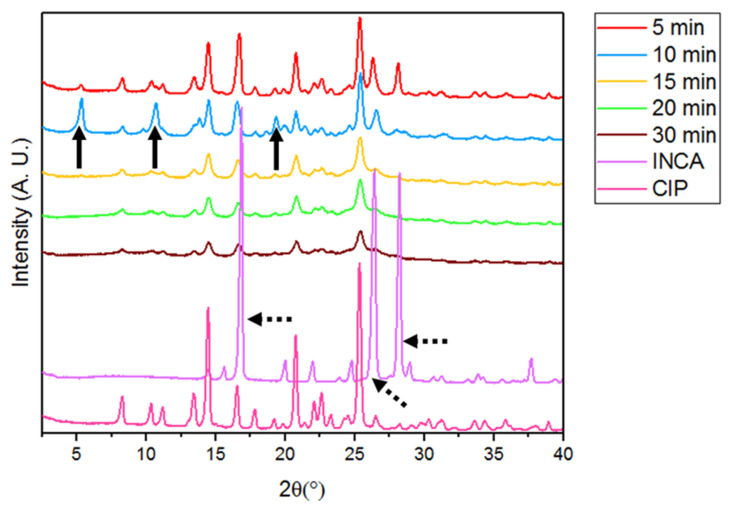
PXRD spectra of all ball milled samples for various duration of times.

PXRD spectra of ball milled samples are shown in Figure 6. The appearance of new peaks at 5.4°, 10.6°, and 19.2° can be seen only in samples that were milled up to 10 min. Interestingly, after 10 min of ball milling, peaks that were associated with cocrystal formation are no longer visible in the spectra. Moreover, the rest of the peaks in these spectra are experiencing a decrease in intensity. This phenomena is most likely correlated with the gradual formation of co-amorphous compound due to the excess mechanical stress applied during the ball milling process [46]. These observations were verified by DSC analysis of ball milled samples.

#### 3.2.2. Differential Scanning Calorimetry (DSC)

**Figure 7:**
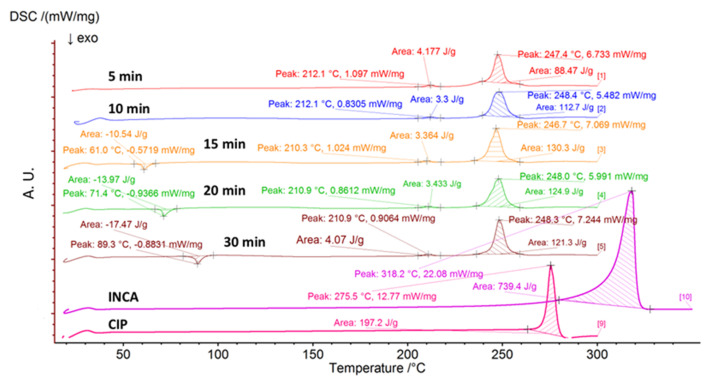
DSC thermogram of ball milled samples for various duration of times.

Similar to the ground samples, two main peaks at approximately 210–212 °C and 246–248 °C were observed in the DSC thermogram of all samples (Figure 7). These peaks are attributed to the presence of the phase transition and melting of cocrystal, respectively. However, an extra exothermic peak at 61 °C, 71.4 °C, and 89.3 °C was present in each of the samples that were ball milled for 15, 20, and 30 min, respectively. Not only did the PXRD spectra confirm the gradual increase in the amorphous content of the samples after ball milling for longer periods but also the presence of exothermic crystallization peaks in DSC thermograms of these samples revealed the recrystallization of a portion of co-amorphous content. The effect of time on the formation of co-amorphous blends has been shown by Lobmann et al. in which they observed a general increase in the formation of co-amorphous blends after ball milling for longer periods of time [47]. Consequently, only 10 min ball milling at 30 Hz would suffice for the formation of cocrystal between the two molecules.

There is a discrepancy between the approximate amorphous content shown in PXRD (Figure 6) and DSC (Figure 7) that needs to be explained. As it can be seen from the PXRD graphs, apparently samples that were milled for 30 min have a substantially high amount of amorphous content, while the DSC graph suggests otherwise. The lower amount of amorphous content in DSC samples is likely due to the transformation of some portions of amorphous content to cocrystal due to the heat during the DSC analysis. In order to prevent this transformation, it is recommended to run the DSC analysis at a much higher heating rate (e.g., 50 or 100 °C) as well and compare the results with the lower heating rate.

### 3.3. Hot-Melt Extrusion

#### 3.3.1. Powder X-ray Diffraction (PXRD)

**Figure 8:**
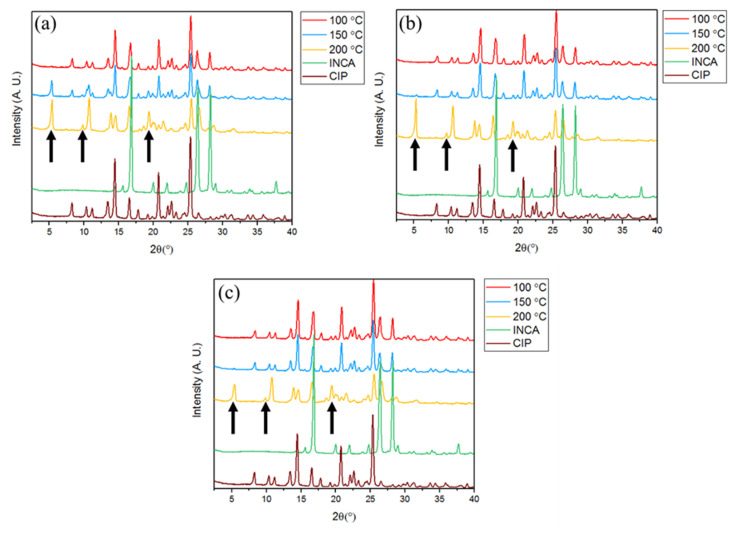
PXRD spectra of samples extruded at various temperature using conveying screw configuration at screw speeds of (**a**) 10 rpm, (**b**) 55 rpm, and (**c**) 100 rpm.

**Figure A1:**
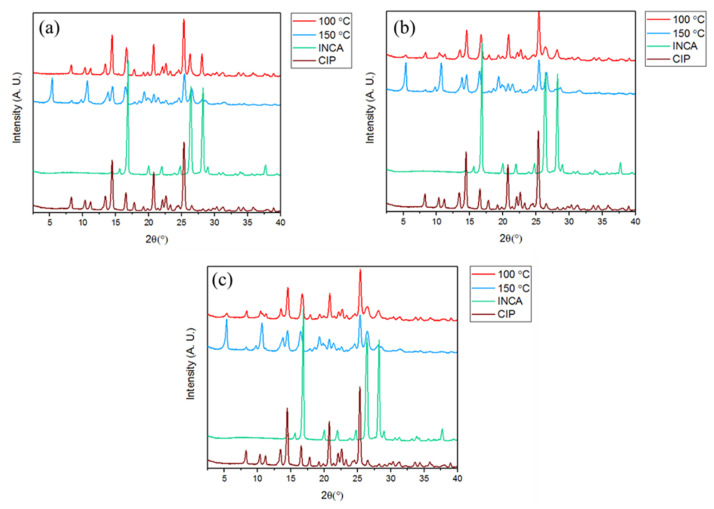
PXRD spectra of samples extruded at various temperature using kneading screw configuration at screw speeds of (**a**) 10 rpm, (**b**) 55 rpm, and (**c**) 100 rpm.

Studying the PXRD graphs of samples produced at various temperatures and screw speeds via two screw configurations—i.e., conveying and kneading—depicts the critical effect of temperature and screw speed on the final cocrystal yield (Figure 8). Overall, regardless of the screw speed, minor cocrystal formation was observed in samples granulated at 100 °C. This is most likely attributed to the absence of an essential intermediate molten phase that could potentially boost the chemical interaction between the two components. However, the lowest screw speed of 10 rpm led to a higher cocrystal formation even at temperatures as low as 150 °C, which is at least 70 °C lower than the eutectic temperature of CIP and INCA based on the binary phase diagram of this model drug system reported in the literature [40]. This is most likely due to the enhanced mixing and wider residence time of powder along the barrel at this screw speed. The presence of extra peaks at 2Theta = 11–11.5° at 150 °C is due to the start of transition of initial precursors to the cocrystal at this specific temperature. These intermediate peaks are not present at 200 °C, which depicts the end of the transition period and the formation of pure cocrystal. As the efficiency of mixing decreases at higher screw speeds, only a negligible portion of cocrystal was formed at 150 °C. Nevertheless, at 200 °C, cocrystal was formed at all screw speeds confirming the critical importance of temperature in cocrystal formation in accordance with the results obtained from statistical analysis. In samples granulated using screw with integrated kneading elements, small portions of cocrystal were formed at 100 °C at all screw speeds. The increase in the intensity of cocrystal characteristic peak at 5.4° by increasing the temperature to 150 °C infers the significant role of temperature in formation of CIP–INCA cocrystal (Appendix A, Figure A1).

#### 3.3.2. Differential Scanning Calorimetry (DSC)

**Figure 9:**
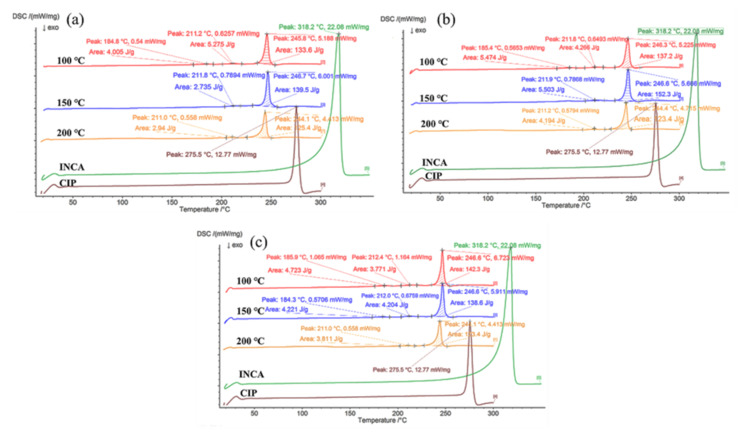
DSC thermograms of extrudates prepared at various temperature and screw speed using conveying screw configuration (**a**) 10 rpm, (**b**) 55 rpm, and (**c**) 100 rpm.

The DSC thermograms of extrudates prepared using conveying screw configuration at various temperatures and screw speeds are shown in Figure 9. In all thermograms, two endothermic peaks at 211 °C and 246 °C were observed. Comparing these peaks with the binary phase diagram of CIP–INCA prepared by A. C. Almeida, confirms that the first peak shows the crystalline phase transition while the second peak is the melting point of the cocrystal [40]. Moreover, a third endothermic peak at 185 °C is present in the samples granulated at 100 °C regardless of the screw speed and samples granulated at 150 °C and 100 rpm. This peak, which is attributed to the physical form transition of the eutectic peak, has only been present in samples with minimal cocrystal formation. The presence of distinctive melting peaks at totally different temperatures compared with the initial materials and the absence of the melting peaks of the initial precursors confirm the formation of a new cocrystal compound between CIP–INCA. In samples that were granulated using screws with kneading configuration (Figure A3), the cocrystal melting peak was detected in all samples due to the higher applied mechanical force during extrusion.

#### 3.3.3. Scanning Electron Microscopy (SEM) and Morphological Analysis

**Figure 10:**
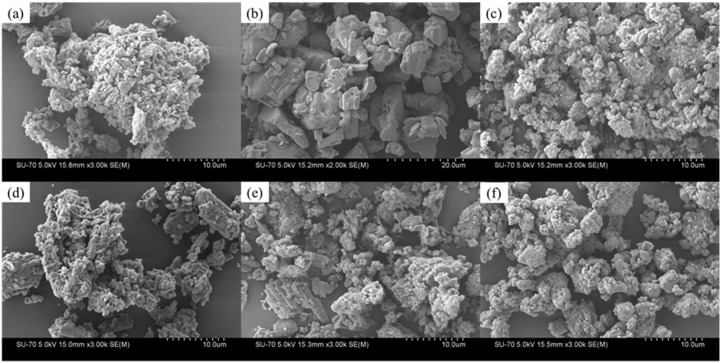
SEM images of the (**a**) CIP, (**b**) INCA, (**c**) sample 10, (**d**) sample 3, (**e**) sample 15, and (**f**) sample 13.

SEM images of the initial materials and four extruded samples under various conditions are shown in Figure 10. Ciprofloxacin is consisted of agglomerates in the range of 10–30 μm consisted of smaller particles. Particles of INCA are in the range of 2–20 μm without any agglomeration. All the extrudates regardless of the process conditions are highly agglomerated particles that are formed by smaller particles. However, slightly large agglomerates are visible in sample 15 which was extruded at 100 °C as the lowest temperature compared with the rest of the samples.

#### 3.3.4. Fourier Transform Infrared Spectroscopy (FTIR)

**Figure 11:**
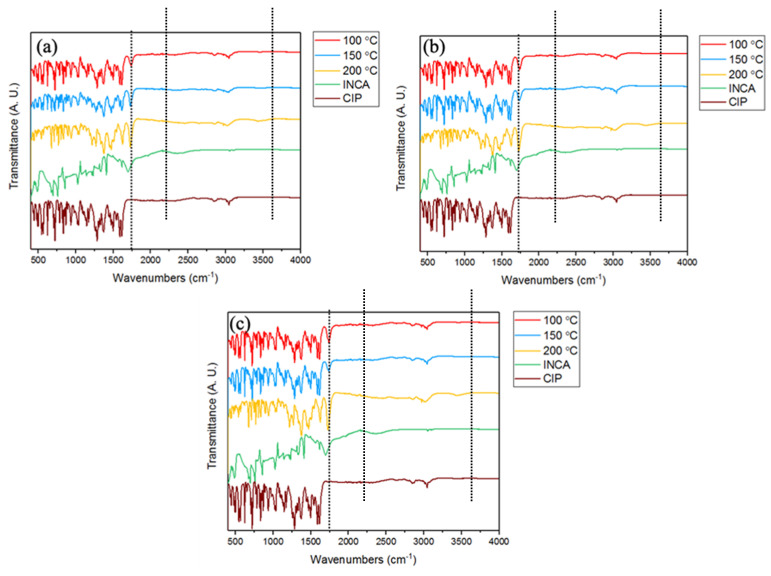
FTIR spectra of samples extruded at various temperatures and screw speeds using conveying screws (**a**) 10 rpm, (**b**) 55 rpm, and (**c**) 100 rpm.

**Figure A2:**
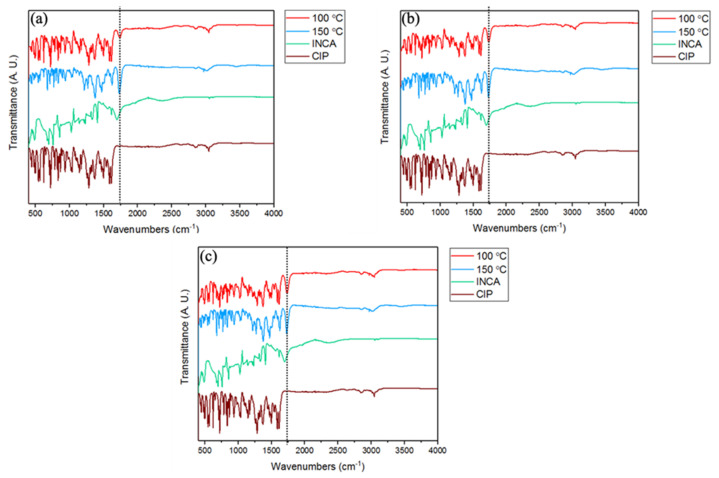
FTIR spectra of samples extruded at various temperatures and screw speeds using kneading screws (**a**) 10 rpm, (**b**) 55 rpm, and (**c**) 100 rpm.

**Figure A3:**
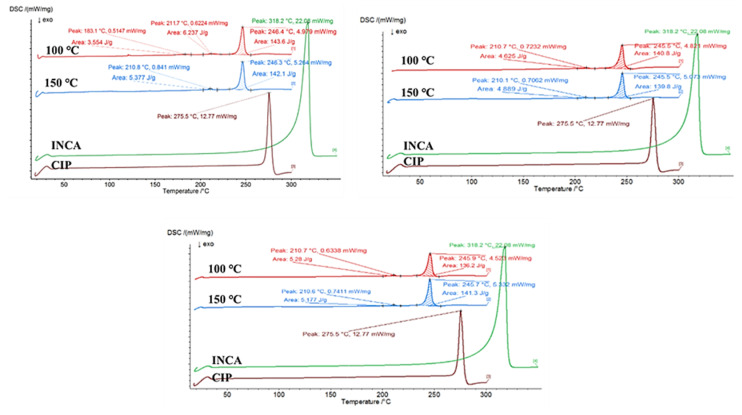
DSC thermograms of extrudates prepared at various temperature and screw speed using kneading screw configuration (**a**) 10 rpm, (**b**) 55 rpm, and (**c**) 100 rpm.

The IR spectra of extruded samples and the initial precursors were obtained in order to identify the possible intermolecular interactions and the extent of cocrystal formation for samples extruded using conveying and kneading screw configurations (Figure 11 and Figure A2). The IR spectrum of CIP was similar to the data available in the literature [41,48]. The presence of strong bands between 1702 cm^−1^ (νC=Ocarbonyl) and 1623 cm^−1^ (δN-Hamine bending) and weak bands in the range of 2580–2680 cm^−1^ that are related to the presence of ν NH^2+^ group confirmed the presence of CIP in the zwitterionic form [49]. Moreover, the peak at 3403 cm^−1^ is related to (νO-Hcarboxylic acid). The INCA spectrum shows the peaks at 3425 cm^−1^(νO=Hcarboxylic acid), 1616 cm^−1^(δN-Hamine bending), and 1716 cm^−1^ (νC=Ocarbonyl) which is in agreement with the available data in the literature [50].

The IR spectra of extruded samples show a blue shift of the νC=Ocaboxyl group of INCA from 1716 cm^−1^ of CO=(νC=O) stretching to 1728 cm^−1^ in cocrystal form, which refers to the possible formation of a hydrogen bond between CIP and INCA molecules. Moreover, the intensity of the peak at (δN-Hamine bending) is gradually decreased by increasing the temperature until it completely disappears from the spectrum of cocrystal at 200 °C. This is an indication of the involvement of N of CIP in a hydrogen bonding. Therefore, it can be concluded that the hydrogen bond between C=O of INCA and N–H of CIP was formed mainly at samples produced at 200 °C. The FTIR spectra of samples extruded using screws with a kneading configuration at 150 °C showed the blue shift in a high intensity peak at 1705 cm^−1^ to 1728 cm^−1^ (Figure A2). The same shift in the peak position could be seen in FTIR spectra of samples extruded at 100 °C; however, this peak had lower intensity compared with its counterpart at 150 °C.

#### 3.3.5. Statistical Analysis

**Figure 12:**
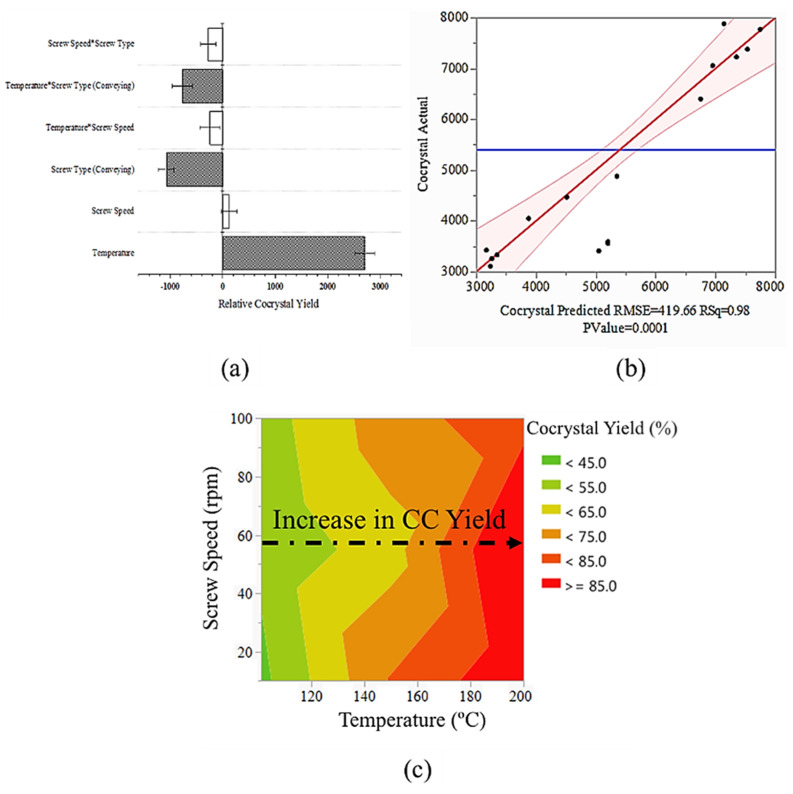
(**a**) Scaled parameter estimates plot with respect to the relative cocrystal yield of hot-melt extrusion process. Intercept estimate = 6276 ± 145. Open bars identify non-significant factors. Shaded bars are the statistically significant factors. Error bars show the estimated standard error for each estimated parameter; (**b**) parity plot shows the goodness of the fit with R^2^ = 0.98; (**c**) contour plot of the effect of screw speed and temperature on the final cocrystal yield of hot-melt extruded samples.

The full factorial ANOVA test revealed the presence of significant factors with substantial effect on the cocrystal yield. It should be mentioned that the series of samples that were extruded with a kneading screw configuration at 200 °C were excluded from the ANOVA analysis and model due to their extremely low physical yield. Based on the results, factors with *p*-values < 0.05 were considered as significant. Figure 12a shows the parameter estimates plot with the most significant factors shown in shaded bars. The scale of each bar depicts the significance of each factor and its effect on the relative cocrystal yield. ANOVA revealed that temperature, screw type, and the interaction of these two factors have impact on the relative cocrystal yield. Surprisingly, the screw speed appeared as a nonsignificant factor with minimal impact. This could be due to the fact that cocrystallization process in this system is more likely controlled by temperature and mechanical force, as opposed to the residence time. The positive impact of kneading configuration on the final cocrystal yield suggests the effect of increased mechanical force due to the presence of kneading elements on increasing the cocrystal yield. Consequently, it is the interaction of these two factors that controls the cocrystallization process of the CIP–INCA system via HME. The data-driven model based on the standard least square regression was in good agreement with the experimental data, as shown in parity plot in Figure 12b.

ANOVA analysis revealed the significant effect of temperature on the final yield of the cocrystallization process. The contour map is a practical and informative tool for deciphering the interrelation of two or more factors and their effect on the studied properties. The contour map in Figure 12c shows the interrelation of temperature and screw speed and their cumulative effect on cocrystallization yield. The cocrystal yield is almost independent of screw speed, with only minimal impact. On the other hand, temperature is the main factor that affects the cocrystal, and the cocrystal yield is directly proportional to the extruding temperature—i.e., higher cocrystal yields have been achieved at higher temperatures regardless of the screw speed.

#### 3.3.6. Dissolution Rate Analysis

**Figure 13:**
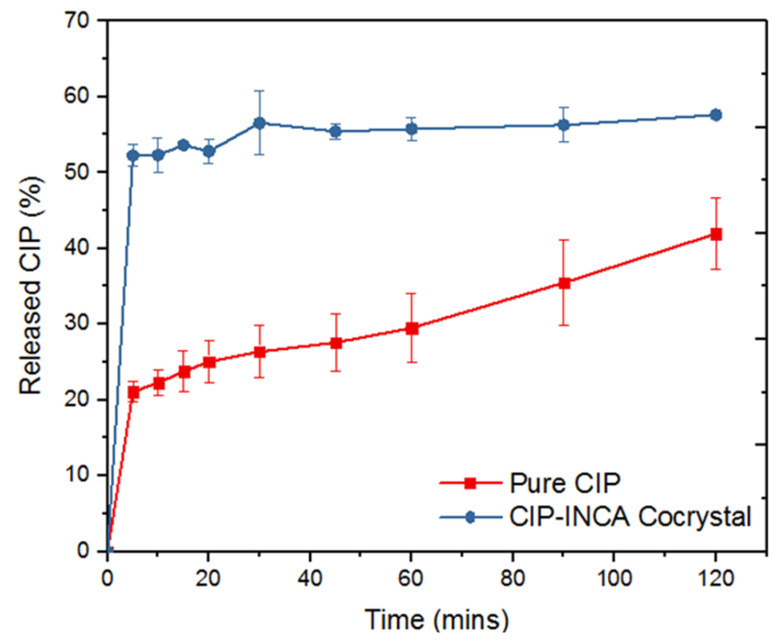
Dissolution rate of pure CIP and CIP–INCA cocrystal in phosphate buffer pH 6.8.

The dissolution rate of an extrudate sample with the highest relative cocrystal yield was measured in a phosphate buffer at pH 6.8 (Figure 13). This range of pH for the buffer was selected as CIP shows the lowest dissolution rate at the pH between 6.1 and 8.7. Additionally, due to the possible changes in the pH of the dissolution media after the dissolution of INCA, a buffer was used instead of pure water as dissolution media [40]. The solubility of ciprofloxacin in phosphate buffer pH 6.8 was reported to be 70 µg/mL and higher than 55 µg/mL, which is the total amount of added API to the dissolution flask. Therefore, the sink condition was maintained during the dissolution test. The cocrystal shows almost a 2-fold increase in the dissolution as soon as 5 min after the start of the test. Despite the faster dissolution rate of the cocrystal formulation compared with that of the pure CIP, the cocrystal formulation dissolution graph reached a plateau after 2 h. This is most likely due to the transformation of dissolved CIP to pure crystalline CIP with lower solubility in dissolution medium. The level of dissolved drug then stabilizes at the same level with only slight increase for the next 115 min until the end of the test, which shows the stability of the dissolved CIP in the dissolution media without any recrystallization or precipitation. The increased dissolution rate of CIP in cocrystal form compared with pure crystalline CIP is mostly likely due to the higher solubility of the INCA component in the dissolution media. This mechanism has been seen in other cocrystals as well [10]. At this stage, the coformer dissolves faster in the dissolution media due to the potential formation of complex bonds between water and INCA molecules. This follows by the disintegration of the crystal structure and release of CIP to the buffer.

## 4. Conclusions

Dry grinding, ball milling, and HME were successfully used for cocrystallization of CIP as a BCS class II API and INCA as coformer. Cocrystal formation was confirmed in all mechanochemical methods used in this study. Higher cocrystal formation was observed by griding the precursors for a longer time. However, ball milling resulted in the formation of a cocrystal at up to 10 min, while at higher milling times, co-amorphous formation was detected. Factorial DoE was employed as a powerful tool for determining the critical process parameters and optimizing the cocrystallization process via HME due to its multivariate nature. ANOVA analysis revealed temperature as the most critical process parameter in cocrystallization via HME. Therefore, the sample extruded at 200 °C showed the highest cocrystal formation. Moreover, it was verified that the kneading screw configuration has a profound effect on the formation of cocrystal even at lower temperatures. Therefore, the most optimized combination of temperature and applied mechanical force was determined in order to achieve the highest possible cocrystallization yield via HME. FTIR analysis confirmed the intermolecular interaction between CIP and INCA, further proving the formation of cocrystal. CIP–INCA cocrystal showed a 2-fold increase in dissolution rate compared with pure CIP. Overall, this study revealed the potential of mechanochemical synthesis, especially HME in combination with a DoE approach for determining the critical process parameters and process optimization.

## Figures and Tables

**Table 1:** Factorial design of experiment and the considered parameters to be studied with their corresponding values.

Number of Experiments	Temperature (°C)	Screw Speed (rpm)	Screw Configuration	Relative CC Yield (%)
1	100	55	conveying	41.3
2	100	10	conveying	43.5
3	150	10	conveying	61.9
4	200	55	kneading	_
5	200	10	kneading	_
6	200	100	conveying	81.2
7	100	55	kneading	51.4
8	100	100	conveying	42.3
9	150	100	Conveying	81.2
10	200	55	conveying	100
11	150	55	conveying	45.6
12	150	55	conveying	48.2
13	150	10	kneading	89.5
14	200	100	kneading	_
15	100	10	kneading	39.5
16	100	100	kneading	56.7
17	150	100	kneading	98.6
18	150	55	kneading	91.7
19	200	10	conveying	93.6

## Data Availability

Not applicable.
